# Concerted bioinformatic analysis of the genome-scale blood transcription factor compendium reveals new control mechanisms†
†Electronic supplementary information (ESI) available. See DOI: 10.1039/c4mb00354c



**DOI:** 10.1039/c4mb00354c

**Published:** 2014-08-18

**Authors:** Anagha Joshi, Berthold Gottgens

**Affiliations:** a The Roslin Institute , University of Edinburgh , Easter Bush Campus , Midlothian , EH25 9RG , UK . Email: anagha.joshi@roslin.ed.ac.uk; b Department of Haematology , Cambridge Institute for Medical Research , Cambridge University , Hills Road , Cambridge , CB2 0XY , UK

## Abstract

Transcription factors play a key role in the development of a disease. ChIP-sequencing has become a preferred technique to investigate genome-wide binding patterns of transcription factors *in vivo*. Although this technology has led to many important discoveries, the rapidly increasing number of publicly available ChIP-sequencing datasets still remains a largely unexplored resource. Using a compendium of 144 publicly available murine ChIP-sequencing datasets in blood, we show that systematic bioinformatic analysis can unravel diverse aspects of transcription regulation; from genome-wide binding preferences, finding regulatory partners and assembling regulatory complexes, to identifying novel functions of transcription factors and investigating transcription dynamics during development.

## Abstract 2

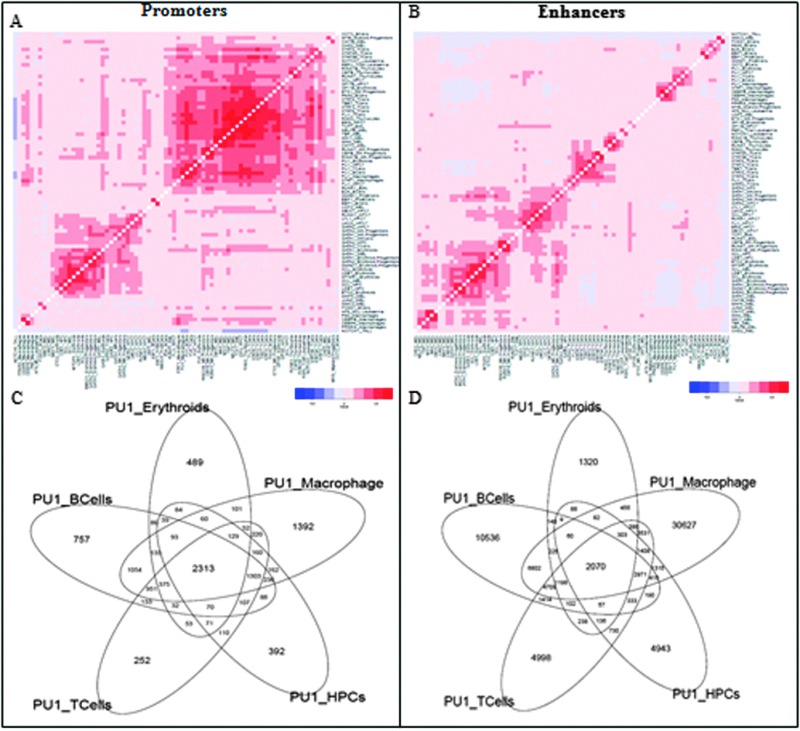
Transcription control insights using blood ChIP sequencing compendium.

## Introduction

The control of cell-type specific gene expression underlies development of all multicellular organisms, and is thought to be achieved through combinatorial interactions of transcription factors with gene regulatory sequences. Moreover, dysregulation of transcription has been widely proven to be a major contributor to human pathologies, with the recent development of small molecule drugs targeting protein interactions between transcriptional regulators generating much excitement.^1,2^

With the interaction between *cis*-regulatory DNA elements and *trans*-acting transcription factors (TFs) representing the fundamental basis of transcriptional control, the delineation of comprehensive collection of regulatory sequences together with knowledge of the TFs bound to them will be essential to gain global insights into transcriptional control mechanisms. Over the past 10 years, chromatin immunoprecipitation (ChIP) followed by microarray (ChIP-chip) or sequencing (ChIP-Seq) have become the most widely used approaches for genome wide identification and characterization of *in vivo* protein–DNA interactions. Due to the rapid drop in the cost of high throughput sequencing, ChIP sequencing has become the method of choice for the generation of high resolution maps of genome-wide protein–DNA interactions in mammalian systems.^3^

To gain a holistic view of transcriptional control during development, it is essential to generate genome scale maps of key transcription factors across multiple cell types. However, generating such genome-scale maps in many different cell types remains a daunting task for individual research groups due to limited human and financial resources. Moreover, each individual TF requires careful validation of antibody reagents, which limits the potential throughput of large-scale initiatives. Indeed, bespoke protocols are often developed by individual groups with specialist expertise, so that published ChIP-Seq studies commonly report binding maps for less than a handful of TFs^4–10^ and only a few larger studies reporting 10 or more factors^11,12^ or a single factor across multiple cell types.^13^ We have previously shown^14^ that unlike gene expression data, ChIP-Seq datasets produced by different laboratories can be readily integrated. This analysis revealed that genome wide transcription factor binding profiles are largely governed by cellular context. We recently reported a TF ChIP-Seq compendium containing 144 publicly available studies pertaining to the mouse blood system.^15^ Using this dataset, here we show how concerted bioinformatic analysis of such a high quality hand-curated compendium can reveal previously unknown aspects of transcriptional control. This includes identification of those TF-bound sites most likely to be functional, prediction of TF interactions and multicomponent complexes, specific functionality of individual TFs and the dynamics of transcriptional regulation during differentiation and development.

## Results and discussion

### Enhancers, unlike promoters, cluster according to the cell type

**Fig. 1:**
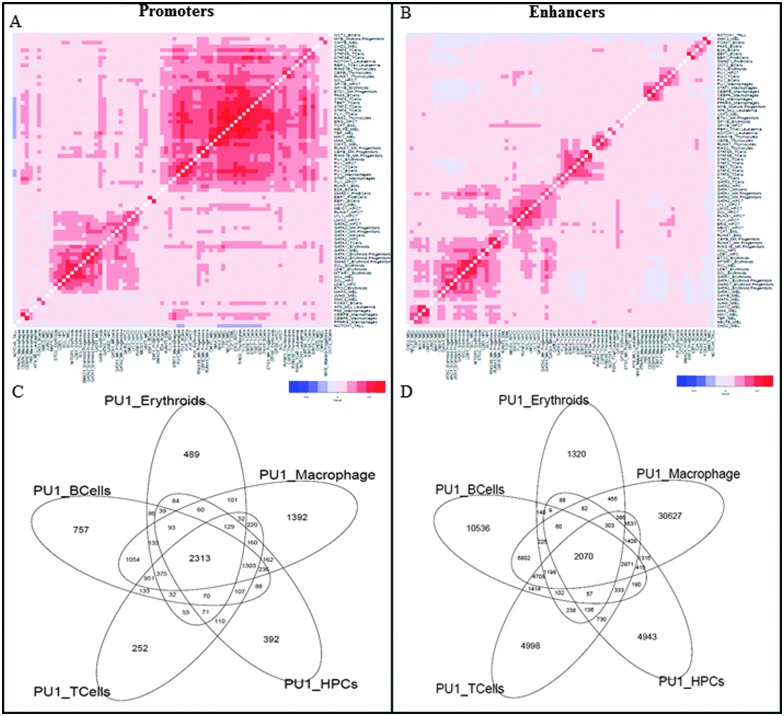
(A, B) Hierarchical clustering of pair-wise peak overlap of all promoters and enhancers across all cell types, red representing positive Pearson's correlation coefficient values and blue representing negative correlation coefficients. (C, D) 5-way Venn diagram of Pu.1 ChIP sequencing data from 5 cell types in promoters and enhancers representing higher overlap in promoters compared to enhancers.

We collected genome-wide binding patterns (peaks) of 144 publicly available murine ChIP-sequencing datasets for 53 transcription factors in 15 major blood lineages and leukemia^15^ to obtain 270 261 regulatory regions with at least one factor binding. We classified peaks into two groups: promoter and enhancer peaks by defining the peaks within 1 kb of TSS as promoter peaks. 7.5% of the total peaks belonged to promoters and all non-promoter peaks were classified as putative enhancers. The hierarchical clustering of enhancers clustered them according to the cell type (Fig. 1B and Fig. S2, ESI†) irrespective of the factors such as Fli1 in hematopoietic progenitor cells (HPC) clustered with other samples in HPCs and Fli1 in T cells clustered with T cell samples. There was an exception of one transcription factor, Pu.1. Pu.1 samples across multiple cell types clustered together.^14^

The promoter regions did not show a strong cell type specific clustering but clustered into two major clusters (Fig. 1A and Fig. S1, ESI†). Cluster 1 consisted of Gata factors across multiple cell types with their known interacting partners such as Ldb1 and Scl/Tal1 and Cluster 2 consisted of a large agglomeration of over 35 samples of multiple factors in diverse cell types. More generally, the observation of lineage-specific pair-wise associations in distal but not promoter regions provides global confirmation for previous suggestions that tissue specific expression is largely mediated by distal elements (Heintzman *et al.*, 2009).

As Pu.1 peaks in both promoters as well as enhancers cluster according to the factor rather than the cell type, we characterized them in more detail. The 5-way Venn diagram of Pu.1 in promoter regions showed a high overlap of binding sites with about 50% of peaks present in all cell types (Fig. 1C) whereas only about 10% of enhancer peaks were present in all cell types (Fig. 1D). This shows that Pu.1 also agrees with the model where promoters mainly drive the cell type invariant while enhancers drive tissue specific expression.

It is well established that transcription factors bind to different interacting partners in a cell type specific manner to drive gene expression.^8^ To check if TFs have distinct interacting partners in promoter and enhancer regions, we calculated *cis*-regulatory motif enrichment in promoter and enhancer regions separately for each factor using HOMER software. The sequence motif of the transcription factor chipped was enriched in both promoters and enhancers in most samples. Most samples also exhibited promoter-specific and enhancer-specific sequence motifs (Fig. S3, ESI†). The GFY-STAF, NRF1 sequence motifs were enriched in promoters of most samples. Only a few motifs were sample specific in promoters such as the Sp1 motif was enriched only in Scl/Tal1 promoter peaks. Sp1 is known to interact with Scl/Tal1 to drive expression of key gene loci such as Kit.^16^ On the other hand, enhancers showed more sample specific motif enrichment. The Ebf1 (early B cell factor) motif is enriched only for Pu.1 enhancers in B cells while MafA (macrophage activating factor) motif is only enriched in Pu.1 enhancers in macrophages.

Taken together, the data support the suggestion that tissue-specificity is a common feature of enhancers rather than promoters.

### Transcription factor gene loci are enriched for peaks

We mapped peaks across 15 blood lineages to their nearest genes resulting in an average of 13.5 peaks per gene. The 19 869 unique gene loci were associated with peaks ranging from a single peak to over 200 peaks. The 726 genes with more than 50 peaks in their gene loci are enriched for functional categories ‘transcription regulation’ (*p*-value: 6.6 × 10^–18^), ‘hematopoiesis’ (*p*-value: 1.9 × 10^–10^) and ‘blood vessel development’ (*p*-value: 8.2 × 10^–8^) demonstrating that hematopoietic regulatory genes have more binding sites in their gene loci. In an individual ChIP-sequencing experiment, most gene loci are associated with only one peak with an average of 1.8 peaks per gene. Genes with more than 5 peaks in their gene locus were enriched for hematopoietic functions. Transcription factor gene loci have an average of 2.5 peaks per gene, in agreement with previously reported suggestions that TF gene loci have a higher number of regulatory elements than average. This difference is statistically significant even after correcting for the gene length (*p*-value: 2.2 × 10^–6^).

It has been suggested that multiple peaks of a TF in a gene locus arise due to cross linking of multiple distant regulatory elements to the promoter, which might explain the lack of a consensus binding motif in many ChIP-seq peak regions.^17^ We calculated the number of enhancer peaks for each factor with and without the presence of a peak at the promoter of a gene and did not observe any bias towards the presence of an enhancer peak with the presence of a promoter peak.

### Candidate regulatory regions bound by multiple factors might be functionally more relevant

**Fig. 2:**
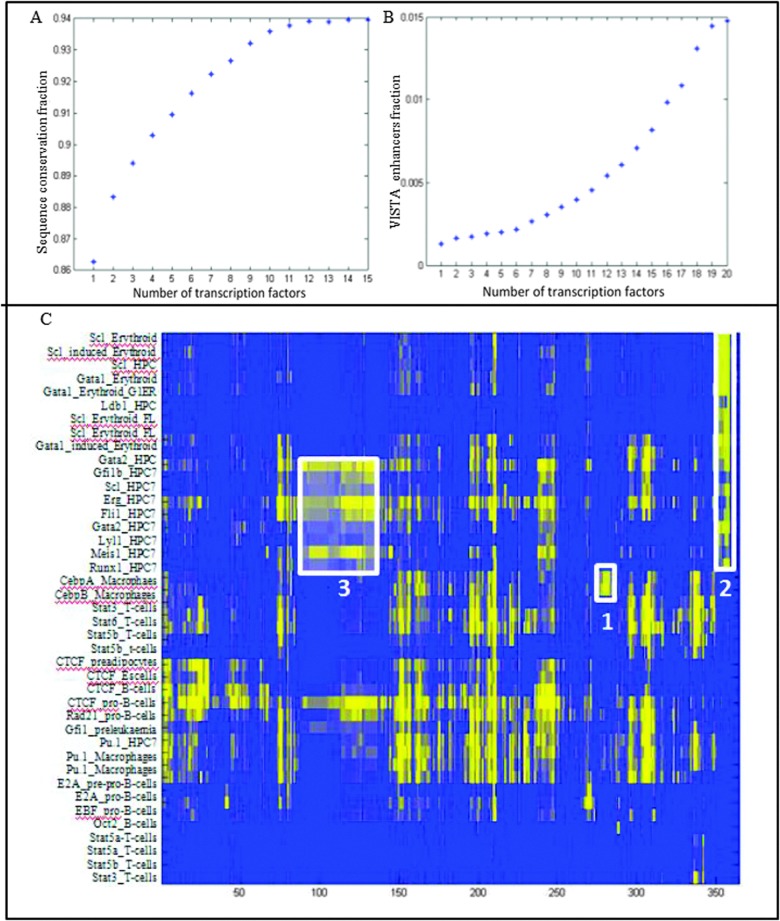
(A, B) Fraction of conserved peaks across human and mouse and fraction of *in vivo* validated peaks (Visel *et al.*, 2007) respectively classified according to the number of transcription factors bound. (C) Heatmap of all ChIP-seq samples against over-represented (yellow) JASPAR motifs showing sequence motifs over-represented in at least one of the samples. Box 1 represents variants of the Cebp motif, box 2 represents variants of the GATA motif, while box 3 represents variants of the homeo-domain motif.

A typical ChIP-sequencing experiment generates millions of reads and hundreds to thousands of peaks. It is widely assumed that not all binding events are of equal functional significance. However, dissecting out functionally important binding events from potentially opportunistic binding events still remains an unsolved problem. Approximately 60% of the 270 thousand peaks of TFs across multiple cell types in blood are bound by more than one factor. We investigated whether the binding of multiple TFs provides any clues towards the functional implications of a binding event. As sequence conservation of a DNA fragment across species is predictive of functionality, we calculated human-mouse sequence conservation scores for all peaks. The sequences underlying peaks bound by multiple factors were more conserved across mammals than those bound by a single factor (Fig. 2A). Moreover, peaks bound by multiple factors were enriched in the VISTA enhancer database (Fig. 2B), a collection of over 700 enhancer regions functionally validated in transgenic mouse assays.^18^ Taken together, these observations suggest that peaks bound by multiple factors might be more likely to be functional. Studies in mammalian cell types indeed have shown that the densely occupied regions tend to lie in the vicinity of genes characteristic of that particular cell type.^11,19^ In addition to the functionality of peaks bound by multiple TFs, it has also been shown that gene loci with multiple binding events are more likely to be functionally significant targets.^20^ Genes bound at multiple locations in most samples are over-represented for developmental processes including ‘muscle tissue development’ and ‘cell fate commitment’, as well as for ‘transcription factor activity’.

### Prediction of new candidate regulatory partners using enriched *cis*-regulatory motifs

Combinatorial transcriptional control is a key aspect of eukaryotic transcription as it provides cell type specificity as well as an ability to integrate multiple signals at a transcriptional level. In order to find over-represented *cis*-regulatory sequence motifs in each ChIP sequencing sample, we used a list of approximately 1300 sequence motifs with known or unknown associated TFs from the JASPAR data-base.^26^ Fig. 2C shows all significantly enriched motifs (*x* axis) for all samples (*y* axis) highlighted in yellow. The enriched motifs are useful in three ways. Firstly, they validate the chipped TF *e.g.* the Cepb motif is enriched in the two samples CebpA and CebpB (Fig. 2C (1)). Secondly, they indicate important binding motifs for a particular cell type, such as enrichment of the GATA motif in HPC7 and erythroid cells (Fig. 2C (2)). Important regulators such as Runx1 and Tal1 are thought to be recruited indirectly to many regulatory regions with the help of GATA factors.^11^ Thirdly and most importantly, new candidate regulatory partners can be predicted, for example a homeodomain box motif is overrepresented only in the binding sites of all factors chipped in hematopoietic progenitor cells (Fig. 2C (3)). Hox proteins, known to play key roles in governing proliferation and differentiation of haematopoietic progenitor cells, can therefore be nominated as new candidate interacting partners with the other blood stem cell factors present in the compendium.

### Transcription factors show preference to a particular genomic location

In order to investigate whether TFs have a preference for specific genomic contexts, we used HOMER^8^ to calculate enrichment with respect to 9 categories defining the gene structure such as 3′ UTR, 5′ UTR, Exon, Intron, Inter-genic, and Promoter regions as well as repeat elements such as LINE, SINE and LTR. All transcription factors were enriched for promoter binding as expected. The components of the Ldb1 complex in erythroid cells were specifically enriched for intronic regions while Chd2 and Smc3 in MEL and Notch1 in T-ALL samples were enriched for 3′ UTR regions (Fig. S4, ESI†). All Pu.1 samples were enriched for LTR repeat elements whereas CebpA and CebpB in macrophages were enriched for SINE repeat elements (Fig. S5, ESI†). Bourque *et al.* ^21^ showed that binding sites of five transcription factors ESR1, TP53, POU5F1, SOX2, and CTCF are embedded in distinctive families of transposable elements which facilitate dynamics in the transcriptional network during evolution such as new locations of CTCF binding generated by SINE repeat element expansion in mammals.^22^ The repeat region enrichment analysis thus provides clues towards how these transcription factors might have gained new regulatory sites during evolution.

Another genomic feature thought to be important for transcription control are CpG islands which facilitate the promoter function by destabilising nucleosomes and attracting proteins that create a chromatin state suitable for transcription.^23^ Rozenberg *et al.* ^24^ observed that the frequency of six TFBS (ETS, NRF1, BoxA, SP1, CRE and E-box) can accurately predict the presence of CpG islands in promoters suggesting that they are structural elements critical for CpG island function. In line with this, transcription factors such as the three ETS factors Erg, Fli1 and Pu.1 were enriched for CpG rich regions. Interestingly, peaks of components of the Ldb1 complex (Gata1, Gata2, Ldb1, Mtgr1 and Scl) occurred significantly less often than expected by chance in CpG rich regions (Table 1).

**Table 1:** Top 5 over-represented and 5 under-represented ChIP-seq samples with peaks in CpG rich regions along with the corresponding *p*-values

#	Sample	Prefer/avoid	*p*-value
1	Erg_HPC7	Prefer	<1 × 10^–256^
2	Fli1_T-cells	Prefer	<1 × 10^–256^
3	Gfi1b_HPC7	Prefer	<1 × 10^–256^
4	Pu.1_B-cells	Prefer	<1 × 10^–256^
5	Rag2_thymocytes	Prefer	<1 × 10^–256^
5	Ldb1_Erythroid	Avoid	3.4 × 10^–4^
4	Gata1_Erythroid_progenitors	Avoid	8.9 × 10^–8^
3	Lmo2_HPC7	Avoid	9.6 × 10^–5^
2	Lyl1_HPC7	Avoid	5.2 × 10^–8^
1	Smad1_Erythroid_progenitors	Avoid	<1 × 10^–256^

Taken together, we found binding biases of transcription factors with respect to genomic locations, repeats and CpG islands. The functional relevance of these observations remains to be investigated.

### TF complexes can be predicted using ChIP sequencing datasets

Physical interaction of TFs is an important aspect in determining tissue specific gene expression, and cooperative binding to DNA may be subject to spatial constraints. For each TF pair, we mapped the sequence motifs to peaks bound by both TFs and calculated the distance between two motifs. We selected motif pairs displaying a specific distance preference in at least two independent ChIP-seq experiments. Importantly, this analysis recovered previously known spacing of 8–10 bps between GATA and E-box motifs involved in binding of Gata1/Scl/E2A/Lmo2 multiprotein complexes.^25^ Of interest, additional preferred pair-wise spacing could be recovered such as 20 bp spacing between the motifs for CTCF and Pu.1. The functional significance of this remains to be explored. The co-ordinate binding between a major fate determining factor such as Pu.1 with a more architectural transcription factor such as CTCF does however provide tantalizing clues as to how interactions between such factors may potentially be involved in stabilizing cell type specific transcription programs. We also find an overlapping joint motif – CANNTGGAW between Scl and ETS factors (Pu.1 and Fli1).

**Fig. 3:**
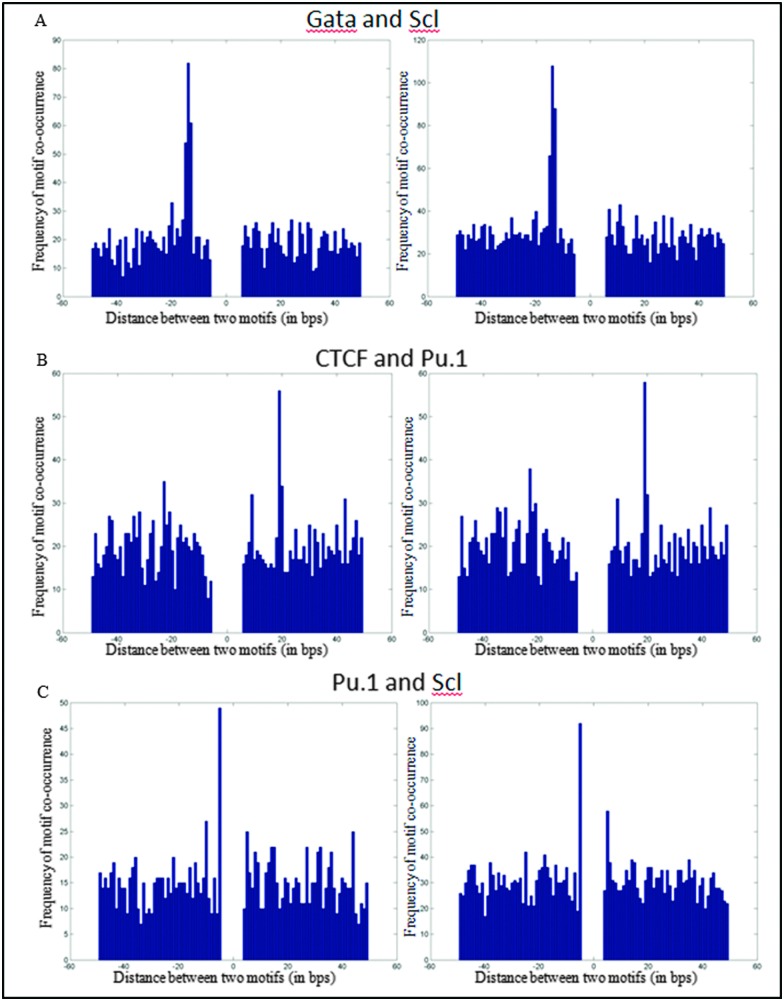
(A) Frequency of the distance between the Scl motif and the GATA motif in peaks occupied by both Gata1/Gata2 and Scl, plotted such that the GATA motif is at position zero. A peak with a 8–10 bps gap between the two sequence motifs is over-represented. (B) Similarly there is a preferred gap of 20 bps between the CTCF and Pu.1 motifs (C). A gap of –1 bp between the Pu.1 and Scl motifs is significantly enriched. Each motif pair was validated by at least two independent ChIP-seq experiments.

To investigate any new motifs showing distance specificity with respect to TF binding sites from our compendium, we calculated distances between each sample and all possible 3 mers (43 = 64 patterns). We found 3 binding distance preferences; the first pattern, GATA and GAT, had a 3/4 bp gap consistent with Gata factors binding as homo-dimers validated by the crystal structure (Bates *et al.*, 2008).^32^ The second pattern, GATA and CTG or GTC, had a 9 bp gap mapping to GATA and a half Ebox binding as a part of the Ldb1 complex. The final pattern, Gfi1b and (A/T)GC, had a 2 bp gap (Fig. 3).

### Lineage priming in progenitor cells

TFs are major determinants of cell fate and lineage choice. However, most lineage determining TFs are expressed across multiple lineages, suggesting that combinatorial interactions are critical in determining cell type specificity. By merging datasets from different studies, the TF ChIP-seq compendium serves as an excellent resource in the study of genome wide binding patterns of the same TF in multiple cell types. Grouping the genome wide binding patterns of Pu.1 in haematopoietic progenitor cells (HPCs) along with two mature cell types (macrophages and B cells) highlights that cell type specific, as well as ubiquitous binding events are present in both promoters and enhancers with ubiquitous binding events being more common in promoters. T and B cells specific functional categories such as ‘lymphocyte activation (*p*-value: 1.9 × 10^–6^)’, ‘immune system development (*p*-value: 5.1 × 10^–4^)’, ‘B cell receptor signalling pathway (*p*-value: 1.2 × 10^–2^)’ are enriched in genes near Pu.1 peaks in HPC7 and B cells and not in macrophages while macrophage specific functional categories such as ‘endocytosis (*p*-value: 2.0 × 10^–5^)’ and ‘inflammatory response (*p*-value: 6.2 × 10^–3^)’ are over-represented in genes near Pu.1 peaks in HPC7 and macrophages and not in B cells. This is a strong indicator of lineage priming in the progenitor cells and therefore provides global confirmation for one of the most hotly debated topics in stem cell biology.

## Methods

The Genome-wide binding patterns of 53 transcription factors in 15 major blood lineages and leukaemia were obtained from ref. 15. Peaks within a 1 kb region from a gene TSS, based on RefSeq gene annotation, were classified as promoter peaks. For each transcription factor pair, the significance of peak overlap was calculated using 1000 randomisations. Human-mouse orthologous regions were downloaded from the MGI database. The overlaps between peaks and human-mouse orthologous regions as well as experimentally validated enhancers in mouse^27^ were calculated using BEDtools.^28^ For the two groups, we calculated whether the pair-wise overlap of promoter and non-promoter peaks was significantly over-represented (red) or under-represented (blue) compared to 100 randomizations. Using HOMER^8^ and based on gene context or repeat elements, peaks were sorted into 9 categories: 3′ UTR, 5′ UTR, exons, introns, intergenic regions, promoters, LINE, SINE and LTR. CpG islands were downloaded from UCSC. A list of transcription factors in mouse was downloaded from RIKEN.^29^ To find distance preferences between pairs of TFs, the sequences for peaks bound by both transcription factors were obtained using UCSC Galaxy and the binding locations of each sequence motif were determined using TFSBsearch.^30^ *cis*-Regulatory sequence motifs were downloaded from the JASPAR library^26^ and the motifs were searched in peaks using TFSBsearch;^30^ over-representation was calculated with respect to 100 random sequence sets of the same number and lengths of real peak sequences. Functional enrichment was calculated using DAVID.^31^ Most analysis was done using Perl, MATLAB and R scripts.

## Conclusions

The advent of next generation sequencing technologies has led to a dramatic shift in modern biological research, where bioinformatic processing and interpretation of large-scale datasets are rapidly replacing data generation as the major bottleneck. Moreover, bioinformatic analysis of genome-scale datasets is often restricted to the particular context of the paper that first reported them, even though the raw data are made publicly available in online repositories. Consequently, a whole potential treasure trove of biological insights remains essentially unexplored.

To ameliorate this situation, progress on two fronts will be vital. Firstly, significant efforts need to be invested into the generation of data integration platforms that facilitate cross-referencing between the multiple independent studies. Secondly, bioinformatic analysis strategies need to be developed to facilitate extraction of novel biological hypotheses from integrated genome-scale resources.

In this paper, we have addressed the latter issue and provided seven examples of bioinformatic analysis that together have allowed us to develop a number of new hypotheses on transcriptional control mechanisms with the potential to transform our understanding of blood cell development. Importantly, both the procedures outlined as well as the take-home messages learned should be readily transferable to the exploitation of ChIP-Seq datasets in other cellular systems, and thus have the potential to significantly advance our understanding of a wide range of both normal and pathological cellular processes.
