# Supplementary material for: Concerted bioinformatic analysis of the genome-scale blood transcription factor compendium reveals new control mechanisms
Source: Mol Biosyst. 2014 Aug 18;10(11):2935–41. doi: 10.1039/c4mb00354c (PMC4263230; doi:10.1039/c4mb00354c)
Supplement: Supplementary file 1 [file MB-010-C4MB00354C-s001.pdf]

Electronic Supplementary Material (ESI) for Molecular BioSystems.
This journal is © The Royal Society of Chemistry 2014

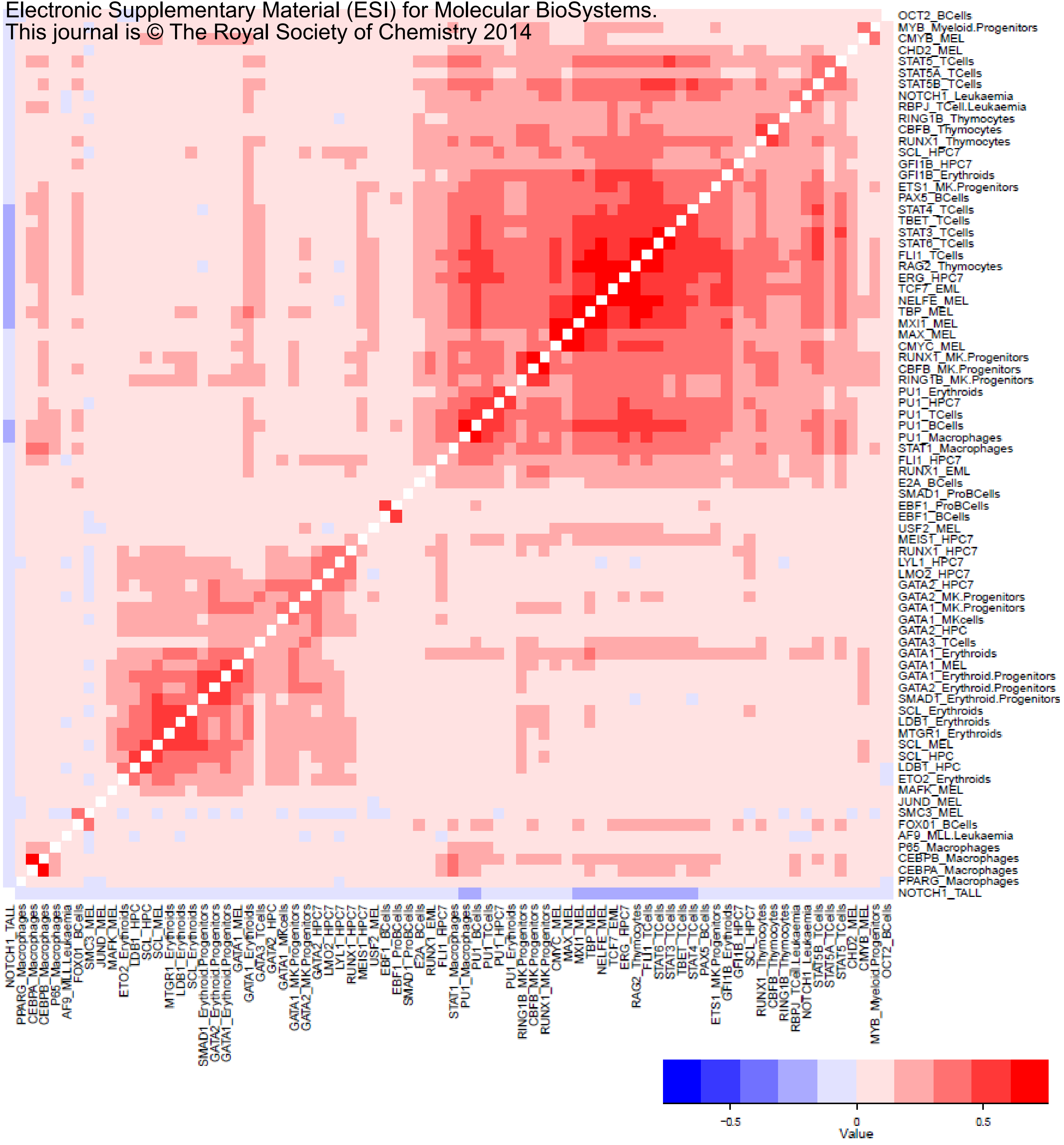

Supplementary Figure 1: Hierarchical clustering of pair-wise peak overlap of all in promoters across all cell types red representing positive Pearson's correlation coefficient values and blue representing negative correlation coefficients

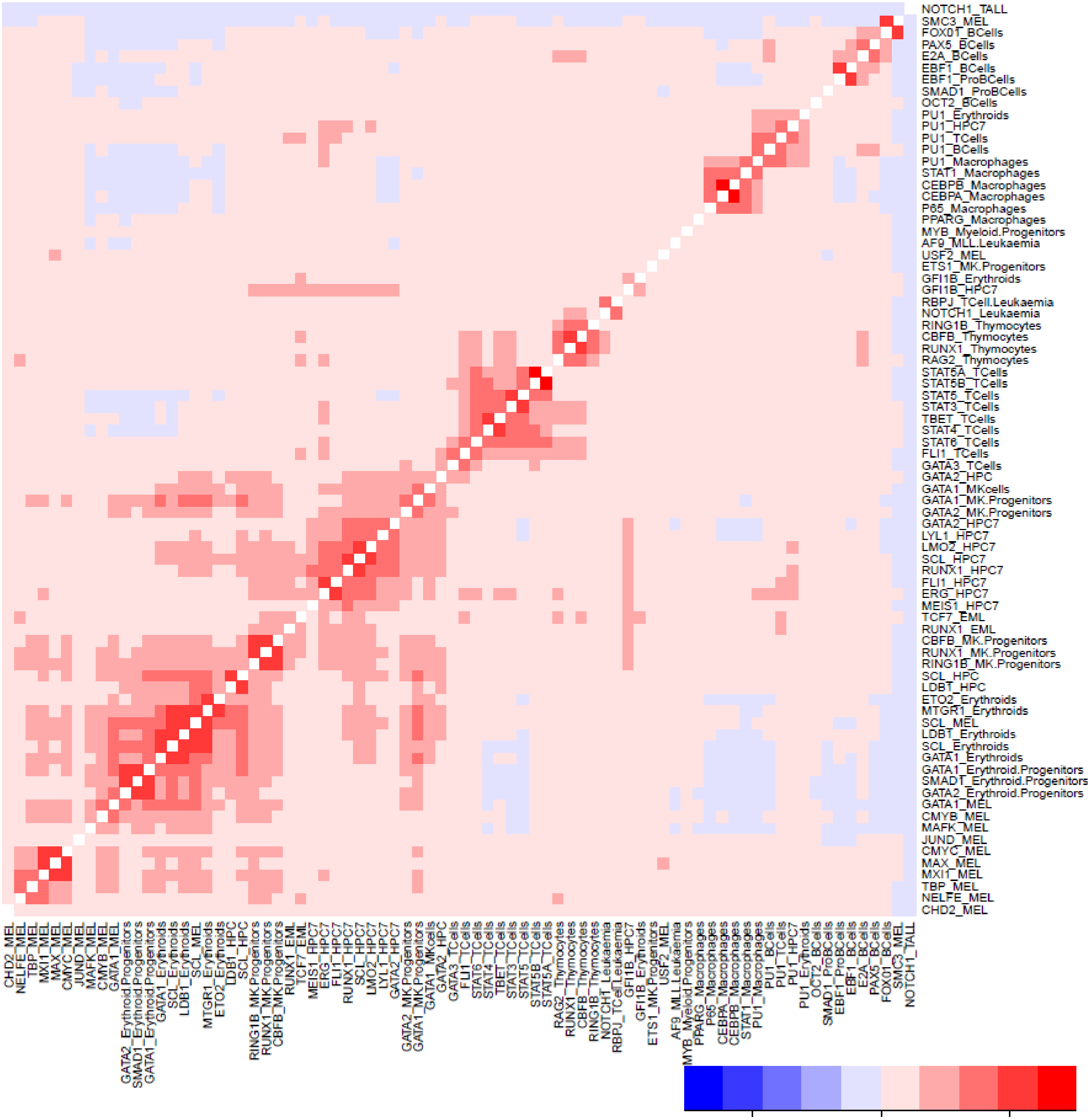

Supplementary Figure 2: Hierarchical clustering of pair-wise peak overlap of all in enhancers across all cell types red representing positive Pearson's correlation coefficient values and blue representing negative correlation coefficients

ChIP-seq sample	Promoter only motifs	Enhancer only motifs
CBFB_MK-Progenitors	E2F4, GFY-Staf, NFY, NRF1	Gata1, Gata2
CBFB_Thymocytes	NRF1	
CEBPA_Macrophages	GFY-Staf, NFY	RUNX2
CEBPB_Macrophages	GFY-Staf, NFY, NRF1	RUNX2
CHD2_MEL	GFY-Staf	Gata4
CMYC_MEL	E2F4, NFY, YY1	
E2A_BCells		E2A-near-Pu1, EBF1
EBF1_BCells	GFY-Staf	
ERG_HPC7	GFY-Staf, NFY, NRF1, YY1,ZNF143| STAF	Foxo1, SCL
ETS1_MK-Progenitors	Atf1, NFY, YY1, ZNF143|STAF	
FLI1_HPC7	GFY-Staf, NRF1, YY1	
FLI1_TCells	GFY-Staf, NFY, YY1	AP-1, Gata1, Gata2, GATA3, Gata4, RUNX1, RUNX2
FOX01_BCells	GFY-Staf	Atoh1, E2A, Myf5, MyoD, MyoG, Olig2, SCL
GATA1_Erythroids	GFY-Staf, NFY, NRF1	RUNX1, RUNX-AML
GATA1_MEL	GFY-Staf	RUNX1, RUNX-AML
GATA2_HPC7		p53, RUNX2
GATA2_MK-Progenitors		RUNX-AML
GATA3_TCells		RUNX1, RUNX2, RUNX-AML
GFI1B_Erythroids	GFY-Staf, NFY, NRF1, YY1	
GFI1B_HPC7		Gata2, GATA3
JUND_MEL		Bach1
MAX_MEL	GFY-Staf, NRF1, YY1	
MEIS1_HPC7		Gata1, Gata2, GATA3, Gata4
MXI1_MEL	E2F4, GFY-Staf, NFY, YY1, ZNF143|STAF	
NELFE_MEL	E2F4, YY1(Zf)	
NOTCH1_Leukaemia	NFY, NRF1, YY1	
OCT2_BCells	GFY-Staf	
PAX5_BCells	E2F4, GFY-Staf, NFY, NRF1, YY1, ZNF143|STAF	Atoh1, EBF1, MyoD, NeuroD1, Olig2, SCL
PU1_BCells	E2F4, GFY-Staf, NFY, NRF1, YY1, ZNF143|STAF	EBF1, RUNX2, Tcf12
PU1_HPC7	GFY-Staf, Klf4, NFY, NRF1	
PU1_Erythroid		Gata1, Gata2
PU1_Macrophages	E2F7, GFY-Staf, NFY, NRF1, YY1, ZNF143|STAF	MafA
PU1_TCells	E2F4, GFY-Staf, NFY, NRF1	
RAG2_Thymocytes	GFY-Staf, NFY, NRF1, Sp1, YY1, ZNF143|STAF	
RBPJ_TCell-Leukaemia	CRE, GFY-Staf, NFY, NRF1	
RING1B_Thymocytes	NRF1	
RUNX1_EML	GFY-Staf, NFY	
RUNX1_MK-Progenitors	GFY-Staf, NFY, NRF1, Sp1	
RUNX1_Thymocytes	GFY-Staf, NFY, NRF1	
SCL_HPC7	NFY, NRF1, Sp1	
STAT1_Macrophages	E2F4, GFY-Staf, NFY, YY1	MafF, Mef2a, Mef2c
STAT3_TCells	GFY-Staf, NFY, NRF1, YY1, ZNF143|STAF	Foxo1, NFAT:AP1
STAT4_TCells	GFY-Staf, NFY, NRF1, YY1, ZNF143|STAF	
STAT5A_TCells	GFY-Staf	
STAT5B_TCells	GFY-Staf, NRF1, YY1	
STAT5_TCells	GFY-Staf, NFY, NRF1	MafA
STAT6_TCells	GFY-Staf, NFY, NRF1, YY1, ZNF143|STAF	Bcl6, Gata1, Gata2, GATA3, Gata4
TBET_TCells	GFY-Staf, NFY, NRF1, YY1, ZNF143|STAF	Eomes, MafK, NFAT:AP1
TBP_MEL	Atf1, E2F4, NFY, Stat3, YY1	ARE, c-Myc, RUNX1
TCF7_EML	GFY-Staf, NFY, NRF1, YY1	Tcf4
USF2_MEL	YY1(Zf)	

sample	genomic_location	prefer/avoid	Pvalue
CBFB_MK-Progenitors	introns	avoid	7.78E-16
CHD2_MEL	introns	avoid	2.30E-29
ETS1_MK-Progenitors	introns	avoid	6.88E-51
FLI1_TCells	introns	avoid	1.83E-35
FOX01_BCells	introns	avoid	2.78E-21
GFI1B_Erythroids	introns	avoid	5.76E-37
MAX_MEL	introns	avoid	7.75E-11
NELFE_MEL	introns	avoid	6.65E-65
NOTCH1_Leukaemia	introns	avoid	3.40E-27
NOTCH1_TALL	introns	avoid	5.18E-25
PU1_Erythroids	introns	avoid	5.22E-16
RAG2_Thymocytes	introns	avoid	1.70E-25
RBPJ_TCell-Leukaemia	introns	avoid	9.99E-24
RING1B_Thymocytes	introns	avoid	1.04E-14
RUNX1_MK-Progenitors	introns	avoid	5.22E-19
SMC3_MEL	introns	avoid	2.06E-91
STAT4_TCells	introns	avoid	5.09E-17
STAT6_TCells	introns	avoid	4.09E-37
TBET_TCells	introns	avoid	3.71E-28
TBP_MEL	introns	avoid	3.15E-47
TCF7_EML	introns	avoid	8.50E-34
CEBPA_Macrophages	introns	prefer	4.78E-11
GATA1_Erythroid-Progenitors	introns	prefer	2.38E-72
GATA1_Erythroids	introns	prefer	4.19E-19
GATA1_MEL	introns	prefer	5.59E-37
GATA2_Erythroid-Progenitors	introns	prefer	9.83E-85
LDB1_Erythroids	introns	prefer	1.70E-12
PU1_Macrophages	introns	prefer	1.58E-20
SCL_Erythroids	introns	prefer	2.28E-26
SCL_HPC	introns	prefer	2.26E-17
SCL_MEL	introns	prefer	3.90E-28
SMAD1_Erythroid-Progenitors	introns	prefer	1.46E-32
CHD2_MEL	utr3	prefer	5.90E-14
NOTCH1_TALL	utr3	prefer	1.49E-74
SMC3_MEL	utr3	prefer	2.25E-23

Supplementary Figure 4: Enrichment within each ChIP sequencing sample for specific genomic location

CEBPA_Macrophages	SINE|MIR	prefer	3.99E-11
CEBPB_Macrophages	SINE|MIR	prefer	4.80E-12
E2A_BCells	GSAT_MM|Satellite|Satellite	prefer	2.49E-11
FLI1_HPC7	RLTR45|LTR|ERVK	prefer	1.84E-69
FLI1_HPC7	ORR1E|LTR|MaLR	prefer	1.85E-17
FOX01_BCells	B3|SINE|B2	prefer	1.49E-76
GATA1_Erythroids	LSU-rRNA_Hsa|rRNA|rRNA	prefer	1.97E-20
GATA2_Erythroid-Progenitors	RLTR18B|LTR|ERVK	prefer	1.46E-11
GATA2_HPC7	HAL1-2a_MD|LINE|L1	prefer	1.53E-16
MAFK_MEL	tRNA	prefer	3.28E-26
PU1_Erythroids	RLTR45|LTR|ERVK	prefer	2.60E-76
PU1_HPC7	RLTR45|LTR|ERVK	prefer	3.82E-47
PU1_Macrophages	ORR1E|LTR|MaLR	prefer	2.17E-16
PU1_TCells	RLTR45|LTR|ERVK	prefer	2.26E-105
PU1_TCells	ORR1E|LTR|MaLR	prefer	1.83E-17
RBPJ_TCell-Leukaemia	RMER21A|LTR|ERV1	prefer	1.23E-17
RBPJ_TCell-Leukaemia	LSU-rRNA_Hsa|rRNA|rRNA	prefer	7.18E-12
RUNX1_HPC7	Satellite	prefer	6.03E-17
SCL_Erythroids	LSU-rRNA_Hsa|rRNA|rRNA	prefer	1.15E-24
SMC3_MEL	SINE	prefer	3.03E-69
STAT5A_TCells	LSU-rRNA_Hsa|rRNA|rRNA	prefer	3.57E-11
STAT5B_TCells	LSU-rRNA_Hsa|rRNA|rRNA	prefer	4.39E-12
TBP_MEL	B2_Mm1a|SINE|B2	prefer	2.13E-68
TBP_MEL	B2_Mm1t|SINE|B2	prefer	8.73E-34
TBP_MEL	B2_Mm2|SINE|B2	prefer	2.02E-23
TBP_MEL	SINE|B2	prefer	3.92E-20
TBP_MEL	tRNA	prefer	2.00E-12

Supplementary Figure 5: Enrichment within each ChIP sequencing sample for specific repeat element
